# Cells Gone Wild: A Case Report on Missed Acute Leukemia and Subsequent Disseminated Intravascular Coagulation in the Emergency Department

**DOI:** 10.5811/cpcem.2022.9.57811

**Published:** 2022-11-11

**Authors:** Onyinyechukwu Okorji, Rachael Kern, Shaylor Klein, Brian Jordan, Kuljit Kaur

**Affiliations:** Jefferson Health - Northeast, Department of Emergency Medicine, Philadelphia, Pennsylvania

**Keywords:** case report, acute leukemia, hyperleukocytosis, disseminated intravascular coagulation

## Abstract

**Introduction:**

Emergency physicians must maintain a broad differential when seeing patients in the emergency department (ED). Occasionally, a patient may have an undiagnosed, life-threatening medical condition not related to the presenting chief complaint. It is imperative to review all ordered laboratory tests and any available previous laboratory values to assess for any abnormalities that may warrant further evaluation.

**Case Report:**

This case report is regarding the missed diagnosis of acute leukemia and subsequent disseminated intravascular coagulation in a 27-year-old male who presented to multiple EDs with the unrelated chief complaint of finger ring entrapment. This patient ultimately succumbed to his illness.

**Conclusion:**

When evaluating patients in the ED, it is important to review any prior available test results for abnormalities, even if the results do not specifically correlate with the chief complaint. Emergency physicians must remain vigilant to avoid missing a critical diagnosis.

## INTRODUCTION

In the emergency department (ED), the breadth of possible diagnoses requires physicians to maintain a broad differential. Potentially missed diagnoses can result in negative patient outcomes; therefore, it is imperative to fully review all available test results, even if not related to the chief complaint. In this case, we discuss a patient presenting with a chief complaint of finger ring entrapment who was subsequently diagnosed with acute leukemia based on his initial complete blood count (CBC) with cell differential. Chart review revealed multiple recent ED visits with abnormal CBCs that had not been addressed. This patient went on to develop disseminated intravascular coagulation (DIC) and ultimately succumbed to his illness.

## CASE PRESENTATION

This case is regarding a 27-year-old male with a medical history of intravenous (IV) opioid use disorder who was brought to the ED by emergency medical services following an assault with a complaint of right-hand pain. No further history was provided by the patient. Upon arrival, he was ill-appearing with vital signs of temperature 36.5° Celsius, heart rate 104 beats per minute, blood pressure 114/58 millimeters of mercury (mmHg), respiratory rate 24 breaths per minute, and oxygen saturation 100% on room air. Physical exam revealed bilateral periorbital ecchymosis, anterior neck ecchymosis, petechial rash on his chest, anasarca, and widespread necrotic wounds. An embedded ring was noted at the base of his right fourth finger with significant surrounding edema, erythema, and necrosis.

Upon chart review of the prior two months, we found that the patient had presented to multiple surrounding EDs with the same chief complaint of right-hand pain with ring entrapment. His first presentation to an outside hospital consisted of unremarkable lab work. The patient left against medical advice (AMA) prior to ring removal. He presented an additional nine times with similar outcomes: lab work would be obtained and the patient would leave AMA prior to ring removal. Thirteen days prior to this case presentation, the patient’s ED workup revealed leukocytosis of 68.2 ×10^9^ cells per liter (L) with 19% blasts and thrombocytopenia of 15 × 10^9^/L, which were not addressed due to the patient leaving AMA.

**Image 1:**
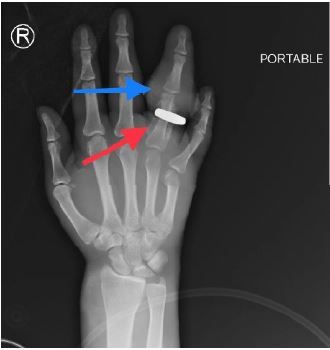
Radiograph of right hand with marked fourth digit swelling distal to the patient’s ring suspicious for tourniquet syndrome. The blue arrow shows soft tissue swelling and the red arrow shows periosteal elevation. Periosteal reaction involving proximal fourth digit phalanges suspicious for osteomyelitis (both arrows).

**Image 2:**
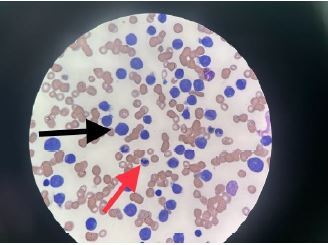
Peripheral blood smear with marked leukocytosis and numerous circulating blasts with monocytic features consistent with acute myeloid leukemia (arrows).

On this current presentation (day zero), a radiograph of his right hand was significant for “tourniquet syndrome” of his right fourth digit with extensive circumferential periostitis (Image 1). The admission CBC was remarkable for a white blood cell count of 157.8 × 10^9^ cells/L (reference range: 4.0–11.0 × 10^9^ cells/L) with blasts of 61% and a subsequent pathology report consistent with acute myelogenous leukemia (AML) (Image 2). See Table 1 for hospitalization laboratory results.

The patient was admitted to the intensive care unit for sepsis secondary to osteomyelitis of the right fourth digit requiring broad spectrum IV antibiotics. Shortly after admission, he developed a change in mental status requiring endotracheal intubation. Computed tomography of the brain did not reveal any acute abnormalities.

**CPC-EM Capsule**

What do we already know about this clinical entity?

*Patients presenting with blast crisis due to acute leukemia require emergent hematology consultation from the Emergency Department in order to initiate treatment and prevent a delay in care*.

What makes this presentation of disease reportable?

*This is a case of an unusual clinical presentation of a medical disease masked by a different chief complaint*.

What is the major learning point?

*To encourage emergency physicians to review and compare current test results with previously available data, despite the urge to only focus on a chief complaint*.

How might this improve emergency medicine practice?

*If physicians review and compare curresnt test results with previously available data, patients can have an appropriate disposition and less delay of care*.

Hematology/oncology was consulted and emergently reviewed the peripheral smear, which revealed blast cells, increased cytoplasmic granules without Auer rods, occasional schistocytes, and a negative Coombs test. Results were consistent with AML, DIC, and auto-tumor lysis syndrome (TLS) due to abnormalities all noted in Table 1. He was emergently treated with rasburicase 6 mg intravenously, hydroxyurea 2 g orally every eight hours, allopurinol 50 mg orally daily, and IV fluids. For treatment of DIC, he received a total of eight units of packed red blood cells, five units of platelets, and one unit of cryoprecipitate. On day three of hospitalization, the patient developed hypotension requiring vasopressor support. He was transferred to an outside hospital for advanced treatment of his AML and succumbed to his illness shortly thereafter.

## DISCUSSION

Leukocytosis is a term broadly used to describe an abnormal elevation of certain cells that originate from the bone marrow. To help guide diagnosis and treatment, one must first distinguish which cell type is elevated. The main cell types measured in the leukocyte count are neutrophils, lymphocytes, eosinophils, monocytes, and basophils, of which neutrophils are the most abundant. Neutrophilia has a wide range of diagnoses ranging from benign to malignant. This is due to upregulation of bone marrow production and/or demargination of existing neutrophils from the endothelium.

Acute elevations can be linked to recent emotional or physical stress, infection, medications, trauma, and smoking. Chronic elevations are the result of inflammatory diseases such as rheumatic disease, inflammatory bowel disease, chronic hepatitis, vasculitis, steroid use, bone marrow stimulation, and congenital diseases. Infection and other forms of acute inflammation will result in leukocytosis with cell counts as high as 25 × 10^9^ cells/L.^1^

When analyzing leukocytosis, cell maturity and the degree of elevation must be taken into consideration. The presence of immature granulocytes and precursors such as blasts and myelocytes are significant abnormal findings and should raise concern for possible malignancy. The morphology of the blast cell may help determine the type of malignancy, which can be AML, promyelocytic leukemia, or other precursor neoplasms.^2^

Hyperleukocytosis is seen with levels greater than 100 × 10^9^ cells/L and should always prompt immediate evaluation for a malignant cause.^1^ Two important lab abnormalities that may be seen with hyperleukocytosis are reverse pseudohyperkalemia and spurious hypoxemia. Although not seen in this patient, reverse pseudohyperkalemia is an in vitro phenomenon in which the potassium concentration is elevated on plasma testing yet normal on serum testing.^3^ This occurs due to intracellular release of potassium as cells undergo lysis due to fragile membranes.^4^ Spurious hypoxemia, which was seen in this patient, is a low partial pressure of arterial oxygen (PaO_2_) noted on blood gas in the presence of normal oxygen saturation. The increased presence of leukocytes results in increased consumption of oxygen, which reflects on the PaO_2_.^5^ In addition, a delay in blood gas processing will result in worsening spurious hypoxemia. This phenomenon is referenced in Table 2. Understanding these lab abnormalities may help mitigate inappropriate or unnecessary treatment of hyperkalemia and hypoxemia. Acute leukemia has a high mortality due to complications including leukostasis, TLS, and DIC.

Tumor lysis syndrome is an oncologic emergency that occurs when the lysis of tumor cells leads to a massive release of intracellular ions such as potassium, phosphorus, and nucleic acids that metabolize to uric acid. This condition is most often diagnosed in patients with leukemia and an exceedingly high white blood cell count.^6^ In this condition, kidney damage occurs via uric acid obstructive uropathy; potassium and phosphorus build-up can cause fatal arrhythmias and hypocalcemia, respectively.

In high-risk patients, allopurinol may be started prior to initiation of chemotherapy to help prevent TLS as it decreases the production of uric acid. Treatment of TLS begins with crystalloid fluids to help expand intravascular volume and to help improve the glomerular filtration rate. Recombinant urate oxidase (rasburicase) may be used to treat hyperuricemia in cases that involve leukemia, lymphoma, and solid tumor malignancy. When possible, it is important to check for glucose-6-phosphate dehydrogenase deficiency prior to administration of this medication. Physicians may also consider additional treatment modalities including sodium bicarbonate infusions for urine alkalinization, hemodialysis, calcium repletion, and febuxostat (xanthine oxidase inhibitor).

Disseminated intravascular coagulation is defined as widespread intravascular fibrin formation in response to increased blood protease activity that overcomes the natural anticoagulant mechanisms.^7^ Vascular endothelial insult results in the formation of systemic microthrombi, which leads to shearing of red blood cells and consumption of coagulation factors and platelets, and can result in hemorrhage. Certain diseases such as promyelocytic leukemia, as noted in this patient, may result in a severe hyper-fibrinolytic state in addition to dysfunction of the coagulation pathway.^7^ Clinical features of DIC include bleeding, thrombosis, shock, acute renal failure, acute liver failure, central nervous system involvement, and respiratory dysfunction. In cases of DIC related to severe sepsis, as seen in this patient, purpura fulminans may be present and are described as large purpuric lesions that result from extensive tissue thrombosis and hemorrhagic skin necrosis.

Testing for DIC consists of coagulation tests, such as platelet count, prothrombin time (PT), D-dimer, fibrinogen, and a peripheral blood smear to assess for the presence of schistocytes. Patients with severe liver disease may exhibit similar lab findings; therefore, lab work should be repeated in 6–8 hours as DIC will exhibit rapid and dramatic changes over a short time frame.^8^

Treatment for DIC begins with determining and correcting the underlying cause. Platelet replacement should be considered when platelet levels are below 50 ×10^9^ cells/L of blood with active bleeding or below 20 × 10^9^ cells/L without evidence of bleeding. Fresh frozen plasma may be transfused to a goal PT and partial prothrombin time less than 1.5 times the normal limit. Cryoprecipitate repletion may be given to maintain a fibrinogen goal of 100–150 mg/dL. Prothrombin complex concentrates and tranexamic acid are generally contraindicated in DIC as they may increase the risk of thrombotic complications.^9^ Extensive thrombosis may warrant anticoagulation therapy with heparin infusion, and those without active bleeding may receive venous thromboembolism prophylaxis with unfractionated heparin or low molecular weight heparin. Finally, activated protein C concentrate should be reserved for cases of severe sepsis with DIC exhibiting purpura fulminans.^10^

## CONCLUSION

When evaluating patients in the ED, it is important to review any prior available test results for abnormalities, even if the results do not specifically correlate with the chief complaint. Our case highlights a patient who presented with finger ring entrapment and a missed diagnosis of leukemia who unfortunately succumbed to his medical illness. We hope this case presentation drives home the point for all physicians to acknowledge and act on all available results in the ED.

## Figures and Tables

**Table 1:** Patient’s laboratory values during hospitalization.

Laboratory values	Dec. 19^2^	Jan. 18	Jan. 29	Jan. 30^3^	Jan. 31	Feb. 1	Feb. 2
Hemoglobin (g/dL)^1^[14.0–17.0]	15.3	10.8	5.1	4.6	7.2	6.9	6.7
White blood cell count (10^9^ cells/L) [4.0–11.0]	9.2	68.2	135.9	157.8	132.8	71.5	60.2
Platelets (10^9^ cells/L) [140–400]	217	15	13	7	21	5	34
Blasts (%) [<0]	-	19	82.9	56	29	38	26
Urea-nitrogen (mg/dL) [10–22]	14	22	51	50	54	69	84
Creatinine (mg/dL) [0.7–1.40]	0.85	1.04	1.98	1.68	2.39	2.68	2.86
Potassium (mmol/L) [3.5–5.0]	3.8	4.3	4.8	4.5	4.4	5.3	5.9
Phosphate (mg/dL) [2.5–4.5]	-	-	-	4.7	7.6	11.7	15.3
Calcium (mg/dL) [8.6–10.0]	9.5	8.6	8.5	7.5	7.7	7.3	6.7
International normalized ratio (INR) [0.82–1.13]	-	1.42	1.75	1.76	1.82	1.87	2.03
Prothrombin time (PT) (seconds) [9.4–13.0]	-	16.4	20.2	21.6	21	21.6	21.8
D-dimer (ng/mL) [<243]	-	-	4539	6562	-	-	10567
Fibrinogen (seconds) [200–393]	-	-	121	109	163	156	137
Lactic acid (mmol/L) [0.5–2.0]	-	-	2.9	2	-	-	5
Lactate dehydrogenase (IU/L) [135–225]	-	-	-	1040	904	911	918
Urate (mg/dL) [3.4–7.0]	-	-	-	16.5	13.1	13.7	15.3
Haptoglobin (mg/dL) [30–200]	-	-	-	39	-	-	-
Reticulocytes (%) [0.5–2.5]	-	-	-	6.2	-	-	-

1 Reference ranges for units

2 Initial presentation with baseline laboratory values obtained

3 Day of presentation of this case presentation (day 0)

*g/dL*, grams her deciliter; *mL*, milliliter; *mg/dL*, milligram per deciliter; *mmol/L*, millimole per liter; *IU/L*, international units per liter; *ng/mL*, nanograms per milliliter.

**Table 2:** Patient’s arterial blood gas on day of admission and expiration.

Laboratory values^1^	Day 0^3^	Day 3
pH^2^ [7.35–7.45]	7.47	7
PaCO_2_ (mm Hg) [34–45]	38	53
PaO_2_ (mm Hg) [83–103]^4^	173	75
Oxygen saturation (%) [95–99]	99.5	94
Bicarbonate (mmol/L) [22–27]	28	13
Base excess (mmol/L) [0–2.4]	3.6	17

1 Laboratory values are as follows: grams her deciliter (g/dL), mL (milliliter), milligram per deciliter (mg/dL), millimole per liter (mmol/L), international units per liter (IU/L), nanograms per milliliter (ng/mL)

2 Reference range for units.

3 Day of presentation of this case study (day 0).

4 Expected PaO_2_ (mmHg) on 100% fraction of inspired oxygen (FiO_2_) is greater than 400.

*PaCO_2_*, partial pressure of carbon dioxide in arterial blood; *PaO_2_*, partial pressure of oxygen in arterial blood; *mm Hg*, millimeters of mercury.
